# Understanding the psychological therapy treatment outcomes for young adults who are not in education, employment, or training (NEET), moderators of outcomes, and what might be done to improve them

**DOI:** 10.1017/S0033291721004773

**Published:** 2023-05

**Authors:** Joshua E. J. Buckman, Joshua Stott, Nicole Main, Daniela M. Antonie, Satwant Singh, Syed A. Naqvi, Elisa Aguirre, Jon Wheatley, Mirko Cirkovic, Judy Leibowitz, John Cape, Stephen Pilling, Rob Saunders

**Affiliations:** 1 Centre for Outcomes Research and Effectiveness (CORE), Research Department of Clinical, Educational & Health Psychology, University College London, 1-19 Torrington Place, London WC1E 7HB, UK; 2 iCope – Camden & Islington NHS Foundation Trust, St Pancras Hospital, London NW1 0PE, UK; 3 ADAPT lab, Research Department of Clinical, Educational and Health Psychology, University College London, Gower Street, London, UK; 4 Let's Talk IAPT – Barnet, Enfield & Haringey Psychological Therapies Service, Barnet, Enfield & Haringey Mental Health Trust, London, UK; 5 Newham Talking Therapies – East London NHS Foundation Trust, Vicarage Lane Health Centre, Stratford, London E15 4ES, UK; 6 Waltham Forest Talking Therapies – North East London Foundation Trust, Thorne House, London E11 4HU, UK; 7 Barking & Dagenham and Havering IAPT Services – North East London Foundation Trust, Church Elm Lane Health Centre, Dagenham, Essex RM10 9RR, UK; 8 Redbridge Talking Therapies Service, North East London NHS Foundation Trust, London, UK; 9 Talk Changes: City & Hackney IAPT Service, Homerton University Hospital NHS Foundation Trust, London, UK

**Keywords:** Anxiety disorders, community mental health services, depressive disorder, psychotherapy outcome research, young adults

## Abstract

**Background:**

To determine: whether young adults (aged 18–24) not in education, employment or training (NEET) have different psychological treatment outcomes to other young adults; any socio-demographic or treatment-related moderators of differential outcomes; and whether service-level changes are associated with better outcomes for those who are NEET.

**Methods:**

A cohort was formed of 20 293 young adults treated with psychological therapies in eight Improving Access to Psychological Therapies services. Pre-treatment characteristics, outcomes, and moderators of differential outcomes were compared for those who were and were not NEET. Associations between outcomes and the following were assessed for those that were NEET: missing fewer sessions, attending more sessions, having a recorded diagnosis, and waiting fewer days between referral and starting treatment.

**Results:**

Those who were NEET had worse outcomes: odds ratio (OR) [95% confidence interval (CI)] for reliable recovery = 0.68 (0.63–0.74), for deterioration = 1.41 (1.25–1.60), and for attrition = 1.31 (1.19–1.43). Ethnic minority participants that were NEET had better outcomes than those that were White and NEET. Living in deprived areas was associated with worse outcomes. The intensity of treatment (high or low) did not moderate outcomes, but having more sessions was associated with improved outcomes for those that were NEET: odds (per one-session increase) of reliable recovery = 1.10 (1.08–1.12), deterioration = 0.94 (0.91–0.98), and attrition = 0.68 (0.66–0.71).

**Conclusions:**

Earlier treatment, supporting those that are NEET to attend sessions, and in particular, offering them more sessions before ending treatment might be effective in improving clinical outcomes. Additional support when working with White young adults that are NEET and those in more deprived areas may also be important.

## Introduction

Depression and anxiety disorders are among the most burdensome diseases worldwide in terms of years of life lost to disability (James et al., 2018; Thornicroft et al., 2017). They are highly prevalent, and result in significant impairment (McManus, Bebbington, Jenkins, & Brugha, 2014; James et al., 2018). The majority of people who have depression or anxiety experience their first episode in adolescence or early adulthood (Kessler et al., 2005). Those with the first onset of depression or anxiety at such a stage of life are at greater risk of experiencing multiple episodes or of having long durations of illness (Buckman et al., 2018a; Curry et al., 2011; Rohde, Lewinsohn, Klein, Seeley, & Gau, 2013), which can have a profound and long-lasting impact on their lives (Monroe, Anderson, & Harkness, 2019; Zisook et al., 2007). As a result, understanding the prognosis and ways in which outcomes might be improved when young adults seek treatment for depression or anxiety may be crucial to lessening the burden of these diseases at the individual and wider societal levels.

Older adults (aged 65 years old and above) appear to have better outcomes in primary care mental health services than those of working ages, particularly among those with anxiety disorders (Saunders et al., 2021). Less is known about younger patients, however, it has been suggested that they might have equivalent outcomes to working-age adults following treatments in primary care (Buckman, Saunders, Stott, et al., 2021; Community and Mental Health team, 2019). Being employed is strongly associated with a better prognosis regardless of the type of treatment given, and after accounting for a number of important clinical indicators of prognosis (Buckman, Saunders, Cohen, et al., 2021). Young people (aged 18–24 years old) are more likely to be in insecure employment than adults of other ages (Chesters et al., 2019; Fiori, Rinesi, Spizzichino, & Di Giorgio, 2016). This was observed to be the case following the global recession from 2008 onwards, and has been related to ‘austerity’ policies enacted during the recession (Chesters et al., 2019; Fiori et al., 2016), but the coronavirus disease-2019 (COVID-19) pandemic is likely to have greatly exacerbated difficulties gaining and maintaining employment, particularly for young adults (Power, Hughes, Cotter, & Cannon, 2020; Probst, Lee, & Bazzoli, 2020). What is more, adolescents and young adults have also had their education severely disrupted during the pandemic (Onyema, 2020). They are therefore more likely to have lost their jobs, to have struggled to find work, and to face financial hardship relative to adults with more years of experience in employment (Chesters et al., 2019).

Young adults not in work or education are often referred to as NEETs (not in employment, education, or training) (Mawn et al., 2017; Office for National Statistics, 2017). A number of studies have found that those who are NEET are more vulnerable to mental health problems and to long-term social and physical health problems (Bäckman & Nilsson, 2016; Gutiérrez-García, Benjet, Borges, Méndez Ríos, & Medina-Mora, 2017; McDaid, Park, & Wahlbeck, 2019; O'Dea et al., 2016). The majority of studies on those who are NEET to-date have been cross-sectional in nature and have sampled from the general population, not those seeking or receiving treatments for their mental health. We do not know therefore, whether those who are NEET have worse outcomes in routine clinical settings than peers of the same age who are in employment, education or training, nor whether this is better explained by any difference in the severity of symptoms or other pre-treatment characteristics that differ between those who are and those that are not NEET. If those who are NEET do have worse outcomes, and these outcomes are not explained by other prognostic factors, it might indicate that they are likely to have poor long-term prognoses as well as worse short-term treatment outcomes (Buckman, et al., 2018a; Buckman, Saunders, Fearon, Leibowitz, & Pilling, 2019).

We also lack knowledge of whether there are important moderators that might lead to differential outcomes for young adults that are and are not NEET. Such knowledge may be of clinical value as it might identify targets for additional or adapted interventions to improve outcomes for those that are NEET. A large study using aggregated data from all primary care and community mental health (Improving Access to Psychological Therapies: IAPT) services in England found that there were five factors that on average, were associated with better treatment outcomes in such services (Clark et al., 2018). In that study, associations were found between: (1) conducting more sessions with each patient; (2) ensuring a higher proportion of patients had a recorded diagnosis; (3) shorter waiting times between referral and starting treatment; and (4) a lower proportion of appointments missed or cancelled, and better treatment outcomes (Clark et al., 2018). A further factor was also associated with better outcomes on average, but only applies at the service level rather than the level of the individual patient, that is, services treating a higher proportion of patients referred to them had better outcomes. When adjusting for these five factors the negative effects of social deprivation were mitigated (Clark, 2018; Clark et al., 2018). We do not know whether such associations may apply to individuals rather than only at the aggregate level, and whether such associations would be found with those that are NEET. Therefore, there is uncertainty whether the same advice given by those authors might be relevant to services seeing those that are NEET in order to mitigate any increased risk of poor outcomes (Clark, 2018; Clark et al., 2018).

This study, therefore, aimed to:

1. To determine whether there are differences in the treatment outcomes, engagement and attrition from psychological therapies for those who are NEET and similar-aged (18–24) peers who are employed, in education, or training, after accounting for pre-treatment differences between the groups including baseline levels of symptom severity, diagnosis, psychotropic medication use, sociodemographics (age, gender, ethnicity, long-term physical health condition status, and area-level deprivation), waiting times before having an assessment appointment and waiting time before starting treatment, and treatment factors such as the type and intensity of treatment, and the number of attended treatment sessions.
2. To determine whether there are sociodemographic or treatment-related moderators (based on gender, ethnicity, indices of multiple deprivations, and the intensity of psychological treatment) of outcomes experienced by those who are and those who are not NEET.
3. To determine whether the four factors highlighted by Clark et al. (2018) that operate at the individual patient level are associated with better outcomes for those who are NEET.

## Material and methods

This study was conducted in accordance with a pre-registered protocol and analysis plan https://osf.io/w2ndr/.

### Dataset and services

Data were provided by all IAPT services that are members of the North Central and East London IAPT Service Improvement and Research Network (NCEL IAPT SIRN) (Saunders, Cape, et al., 2020). These UK National Health Service (NHS) primary care and community-based mental health services deliver psychological therapies for adults with common mental health problems. They offer a range of low-intensity (LI) treatments such as guided self-help, and formal high-intensity (HI) psychological interventions such as cognitive behaviour therapy or counselling, all delivered utilising a stepped-care model in line with national guidelines and evidence-based practice [see (Clark, 2018) for more details]. In IAPT services problem descriptors are used to identify the main presenting problem which will be the focus of treatment. These are based on diagnostic criteria in ICD-10 (World Health Organisation, 1992). The problem descriptor is not necessarily the most severe or the only diagnosis a patient may present with, but is the agreed focus of treatment. The choice of treatment is made jointly between the patient and IAPT clinician, although only those treatments recommended in clinical guidelines for the specific diagnosis are offered (Clark, 2018). All therapists are trained to deliver treatments in line with evidence-based protocols, completing a training course commensurate with the type of treatment they will be delivering in order to work in IAPT (e.g. a diploma in LI CBT). Trainees on such courses also offer treatment in IAPT under the close supervision of trained therapists.

### Participants

A retrospective cohort was formed from all patients aged under 25 years old, whose episodes of care within any of the eight participating IAPT services ended between 1^st^ August 2008 and 1^st^ August 2020 and who received at least two treatment sessions. There is no consensus on the age boundaries for young adulthood, here we used the minimum age of adulthood in the UK and the point at which someone may access adult mental health services (18 years old) and the maximum cut-off for young adulthood adopted by the World Health Organisation and used in many countries around the world (24 years old) (Walker-Harding, Christie, Joffe, Lau, & Neinstein, 2017). Those who were NEET were defined as participants who self-reported that they were not in any type of paid employment, full-time or part-time studies, or vocational training. Those who self-reported being in part-time or full-time employment, education or training (i.e. those who were not NEET) formed the comparison group. Patients were excluded from these analyses if they did not fit into either of these groups (e.g. if they were in voluntary employment only), if they did not report their employment status, or if they had a diagnosis for which there is no recommended evidence-based treatments in IAPT services (Clark, 2018) such as schizophrenia, bipolar disorder, or alcohol dependency. In addition, those who did not have at least two treatment sessions in their episode of care with the IAPT services, as well as those who were not scoring above the clinical thresholds on either the measures of depression or anxiety at their initial assessment (see Table 1 and the Outcomes section below), were excluded from these analyses in line with national reporting of IAPT services (Community and Mental Health team, 2019).

**Table 1.** Available data and measures

Data Item	Questionnaire	Information on measurement
Employment status	N/A	All patients are asked their employment status from a list of ‘Employed’, ‘Unemployed’, ‘Student’, ‘Long-term sick’, ‘Homemaker’, ‘Not seeking work’, ‘Volunteer’, ‘Retired’.
Depression	Patient Health Questionnaire 9-item (PHQ-9; (Kroenke, Spitzer, & Williams, 2001))	To measure symptoms of depression, scores of 10 or above indicate clinical caseness for depression, and a reduction of 6 or more points is used to indicate reliable improvement.
Anxiety	The Generalised Anxiety Disorder Scale 7-item version (GAD7; (Spitzer, Kroenke, Williams, & Löwe, 2006))	To assess generalised anxiety symptoms, a cut-off of 8 or higher is used for caseness and 4 or more for reliable change. Alternative ‘anxiety disorder-specific measures’ (ADSMs) are used when specific anxiety disorders are identified as the ‘problem descriptor’ (Clark, 2018), for example, the Social Phobia Inventory (Connor et al., 2000) for use when a social anxiety disorder is identified. When present, these ADSMs are used to calculate outcomes instead of the GAD-7. The full list of ADSMs alongside the service thresholds for caseness and reliable change is presented in Appendix A and further details are available in the annual reports on the UK IAPT programme (NHS Digital, 2019).
Personal functioning	The Work and Social Adjustment Scale (WSAS) (Mundt, Marks, Shear, & Greist, 2002)	Measures personal functioning in relation to: ‘ability to work’, ‘home management’, ‘social activities’, ‘private leisure activities’ and ‘close relationships’ (domain score range, 0-8). The WSAS item on the ‘ability to work’ is routinely recorded as ‘not applicable’ for individuals not in employment, as a result, this item was excluded from the analyses. As there is no validated total score for the WSAS removing item 1, results are presented separately for each of the domains in items 2-5.
Phobic anxiety	The IAPT Phobia Scales (IAPT National Programme Team, 2011)	Consist of three questions each assessing the degree of avoidance of certain situations related to different types of phobic anxiety - agoraphobia, social phobia, and specific phobias
Problem descriptor	N/A	The services collect data on each patient's problem descriptor (ICD-10 code), representing a probable or confirmed diagnosis based on ICD-10 diagnostic criteria (World Health Organisation, 1992), in order to match patients to evidence-based treatments. We categorised problem descriptors following conventions from previous studies that used similar data (Buckman et al., 2018b; Saunders et al., 2019): depression; mixed anxiety and depression; GAD; obsessive-compulsive disorder; post-traumatic stress disorder; phobic anxiety and panic.
Demographics	N/A	Self-reported gender at point of referral, age, ethnicity (based on UK census codes ‘White’, ‘Mixed’, ‘Asian’, ‘Black’, ‘Chinese’ and ‘other’), sexual orientation, and index of multiple deprivations (IMD) ranks and deciles were available in the dataset. IMD (Department for Communities and Local Government, 2015) measures the relative deprivation of geographical areas (of approximately 1500 residents each) in England. The IMD ranks each area from the most to the least deprived, based on a weighted composite index score made up of deprivation scores in seven domains: income, unemployment, education, health and disability, crime, barriers to housing and services, and quality of the local environment. The ranks are then split into deciles such that the first decile (i.e. = 1) represents the most deprived 10% of areas in the country and the tenth decile ( = 10) represents the least deprived 10% of areas. For this study, due to small numbers in some deciles, the ranks were also split into tertiles, such that being in the first tertile means an area is in the one-third of areas that are most deprived, and being in the third tertile means an area is in the one-third of areas that are least deprived nationwide.
Long-term health conditions	N/A	All patients are asked whether or not they have any long-term physical health condition (LTC). The type of condition was not available in the dataset.
Medication	N/A	At every clinical contact, the services routinely record the psychotropic medication(s) status of their patients in three categories: prescribed and NOT taking, prescribed and taking, and not prescribed.
Treatment factors	N/A	The number of ‘Low Intensity’ (LI) and ‘High Intensity’ (HI) treatment sessions received; the main intensity of treatment received recorded as LI if a patient had more than two LI treatment sessions and less than two HI treatment sessions, or recorded as HI if more than two HI sessions and fewer than two LI sessions. The main type of treatment (e.g. for HI treatments: CBT, Counselling, Interpersonal Psychotherapy, and for LI treatments: Guided Self-Help, Computerised CBT, LI Group CBT, Structured Exercise). The number of days between referral to the service and assessment, the number of days between the assessment and first treatment session, and days between first and last treatment session. Days between referral and sessions, days between assessment and first treatment session, and first and last treatment session were winsorised at the top 99% due to a limited number of extreme values.
Cohort Factors	N/A	The year and month of the first treatment session
Service Factors	N/A	The NHS Trusts and Services from which data were received.

#### Measures

The services routinely collect outcome measures of depression and anxiety symptoms at each clinical contact, as well as a measure of work and social functioning with approximately 99% coverage in pre-post treatment data on these measures (Clark, 2018). Table 1 presents these self-report measures and additional data items that were included in the analyses.

## Data analysis plan

### Outcomes

#### Primary

The primary outcome was ‘Reliable Recovery’ defined (based on national reporting of outcomes in IAPT services) as achieving reliable change on either the PHQ-9 or GAD-7 [or another Anxiety Disorder Specific Measure (ADSM) which replaces the GAD-7 when the diagnosis is of an anxiety disorder other than generalised anxiety disorder (GAD)], or both, **and** moving from ‘caseness’ before treatment on either the PHQ-9 or the GAD-7 (or ADSM) to below caseness on **both** measures following treatment (Community and Mental Health team, 2019). The thresholds for caseness on the PHQ-9 and GAD-7 are scores of ⩾10 and ⩾8 respectively, and reliable change is defined as a reduction of ⩾6 points on the PHQ-9 or ⩾4 points on the GAD-7. See online Supplementary Table S1 for caseness and reliable change thresholds for the ADSMs.

#### Secondary

The following secondary outcomes were:

- Reliable Improvement: achieving reliable change on either the PHQ-9 or GAD-7 (or other ADSM), or both (with thresholds defined in the ‘Reliable Recovery’ section above).
- Deterioration: a reliable increase in symptoms on any symptom-based outcome measure (by the same magnitude as those used to determine reliable improvement above).
- Engagement: defined as the proportion of treatment sessions offered to each patient that the patient attended. Sessions cancelled by the service/clinician were not counted in this outcome.
- Attrition: defined as whether or not the reason for a patient's episode of care ending was reported to have been due to the patient dropping out of treatment prior to the planned ending, after receiving two or more treatment sessions. Patients who declined treatment or were referred on to other services were excluded from these analyses.

### Confounders

Potential confounding factors were those variables outlined in Table 1, including: (1) clinical factors comprised of symptom measure scores (for depression, generalised anxiety, work and social functioning, and phobic anxiety), diagnosis (or ‘problem descriptor’), and medication status; (2) pre-treatment demographics (age, gender, ethnicity, long term condition status, and area-level deprivation based on IMD deciles or tertiles); (3) treatment-related factors including waiting times from referral to assessment, and from assessment to starting treatment, the number of LI and HI sessions attended, and the main type and intensity of treatments; (4) cohort factors including the year and month of the first attended treatment appointment; and (5) service-related factors based on the NHS Trust and services that data were collected in.

### Potential moderators

These included self-identified gender, ethnicity, deprivation [tertiles of the Index of Multiple Deprivation (IMD) rank at the lower-layer super output area (LSOA) level], the main intensity of treatment (LI or HI) (see Table 1 for how defined). The four factors that can be assessed at the level of the individual patient from the Clark et al. (2018) study were also assessed: the number of attended therapy sessions; the number of sessions cancelled or missed by the patient; whether or not a diagnosis was recorded; the number of days between referral and starting treatment, and an extension to that, whether or not patients waited less than 21 days to have their first appointment.

### Data handling and data management

#### Missing data

Missing data on continuous variables that were not systematically missing (also known as missing by design) were imputed using multiple imputations with chained equations with the ‘ICE’ package (Royston & White, 2011) in Stata (StataCorp, 2019). Imputation models included all continuous variables listed in Table 1 and were run to give 50 imputed datasets as per our pre-registered protocol, whereby only variables with less than 50% missingness would be imputed. Missing data on categorical socio-demographic variables were given a ‘missing’ code to allow these participant cases to be used in analyses (i.e. not removed due to list-wise deletion), whilst acknowledging the missing information status on the variable. The effect of the imputation was checked in sensitivity analyses run with complete data only.

### Plan of analysis

To compare baseline characteristics of those who were and those who were not NEET *t* tests were used to explore differences in means of continuous variables between groups, and chi-square tests for categorical variables.

To investigate associations between psychological therapy outcomes and NEET status, a series of regression models were constructed with each outcome listed above (logistic models were fitted for binary outcomes and linear models for continuous outcomes). We started by modelling crude effects in univariable models then added the confounders listed above in order from 1 (clinical factors) to 5 (service-related factors), sequentially, in separate models to calculate adjusted effects. Multilevel regression models were also fitted with random effects for service-level clustering. If the associations of NEET status with the outcomes differed considerably between the multilevel and single-level models, adjusted for all confounders, multilevel modelling would have been used for the adjusted models too. This is a slight deviation from the pre-registered protocol in which we stated this would be conducted in unadjusted models. As there were no differences of note between these modelling approaches, the simpler, single-level models were retained and used for the analyses presented here.

Moderators were explored by fitting interaction terms in the fully adjusted models. In addition, the four factors highlighted by Clark et al. (2018) were assessed in a subgroup analysis of those who were NEET only, to determine the associations between those factors and each of the outcomes listed above among those who were NEET.

### Ethical approvals

NHS ethical approval was not required for this study (confirmed by the Health Research Authority July 2020, reference number 81/81). The data were provided by the IAPT services for evaluation as part of a wider service improvement project conducted in accordance with the procedures of the host institution and the NHS Trusts which operate the IAPT services (project reference: 00519-IAPT).

## Results

### Descriptive statistics

In this analytic sample of 20 293 adults aged under 25 years old, 4608 (22.7%) self-reported to be NEET by virtue of them not being in employment, education, or training at the point of their baseline assessment session with the services. See online Supplementary Fig. S1 for participant flow with details of exclusions. Those that were NEET were more likely to identify as men than those that were not NEET (34% compared to 26.4%), more likely to identify as of Mixed, Asian, or Other ethnicities, and less likely to identify as Black, White, or Chinese. Young adults NEET were somewhat more likely to identify as heterosexual than those that were not NEET, and were more likely to live in socially deprived neighbourhoods (see Table 2). There were no differences in the mean age between the groups. Those who were NEET were more likely to report being prescribed psychotropic medication, were more likely to have a diagnosis of depression or PTSD, and were less likely to have a diagnosis of a GAD, compared to those that were not NEET. On average, those who were NEET had higher scores across all symptom measures pre-treatment and on the work and social adjustment scale, were more likely to report having a comorbid long-term physical health condition, and waited longer between both referral and assessment and assessment and treatment, than their not NEET peers. This is commensurate with the fact that those who were NEET were more likely to have HI therapy as their main treatment intensity, and to have had fewer LI treatment sessions.

**Table 2.** Comparison of baseline descriptive statistics between those who were NEET and those who were not NEET

		NEET	Not NEET	Comparison
Baseline Characteristics	Category	*N* (%) or Mean (s.d.)	*N* (%) or Mean (s.d.)	*t* test or chi-square *p* value
Overall Sample	Total	4608 (22.71)	15685 (77.29)	**20293**
*Socio-demographics*				
Gender	Male	1566 (34)	4137 (26.4)	<0.001
	Female	3019 (65.5)	11449 (73)	
	Missing	23 (0.5)	99 (0.6)	
Ethnicity	White	2581 (56)	9268 (59.1)	<0.001
	Mixed	449 (9.7)	1240 (7.9)	
	Black	495 (10.7)	1958 (12.5)	
	Asian	676 (14.7)	1688 (10.8)	
	Chinese	18 (0.4)	198 (1.3)	
	Other	177 (3.8)	451 (2.9)	
	Missing	212 (4.6)	882 (5.6)	
Age		21.62 (1.88)	21.67 (1.93)	0.11
IMD Decile	1	639 (13.9)	1346 (8.6)	<0.001
	2	1463 (31.7)	4174 (26.6)	
	3	951 (20.6)	3330 (21.2)	
	4	493 (10.7)	2001 (12.8)	
	5	364 (7.9)	1521 (9.7)	
	6	253 (5.5)	1213 (7.7)	
	7	155 (3.4)	746 (4.8)	
	8	133 (2.9)	645 (4.1)	
	9	62 (1.3)	290 (1.8)	
	10	20 (0.4)	120 (0.8)	
	Missing	75 (1.6)	299 (1.9)	
*Clinical Characteristics*				
Psychotropic Medication Status	Prescribed and Not taking	405 (8.8)	1237 (7.9)	<0.001
	Prescribed and Taking	1597 (34.7)	4632 (29.5)	
	Not prescribed	2311 (50.2)	8950 (57.1)	
	Missing	295 (6.4)	866 (5.5)	
Diagnostic Category	Depression	1741 (38.4)	5432 (35.4)	<0.001
	Mixed Anxiety & Depression	279 (6.2)	760 (5)	
	GAD	346 (7.6)	2213 (14.4)	
	OCD	102 (2.2)	419 (2.7)	
	PTSD	265 (5.8)	355 (2.3)	
	Social Phobia	254 (5.6)	798 (5.2)	
	Other Phobia or Panic Disorder	275 (6.1)	845 (5.5)	
	Anxiety Disorder Not Otherwise Specified	70 (1.5)	249 (1.6)	
	Missing	1204 (26.5)	4259 (27.8)	
PHQ-9 Score		16.51 (5.23)	14.86 (5.31)	<0.001
GAD-7 Score		14.44 (4.40)	13.78 (4.26)	<0.001
WSAS Item 2 Score		3.74 (2.54)	3.39 (2.33)	<0.001
WSAS Item 3 Score		4.91 (2.50)	4.33 (2.26)	<0.001
WSAS Item 4 Score		3.81 (2.67)	3.54 (2.45)	<0.001
WSAS Item 5 Score		4.45 (2.52)	4.18 (2.37)	<0.001
Agoraphobia Score		3.73 (1.07)	2.38 (0.84)	<0.001
Social Phobia Score		4.03 (2.64)	3.26 (2.42)	<0.001
Specific Phobia Score		2.78 (2.88)	2.16 (2.54)	<0.001
Long-term Health Condition	No	2954 (64.1)	10699 (68.2)	<0.001
	Yes	756 (16.4)	2150 (13.7)	
Days between referral and assessment		25.48 (30.83)	23.95 (26.34)	0.002
Days between assessment and entering treatment		78.81 (84.90)	73.25 (78.60)	<0.001
*Treatment related variables*				
Main Treatment Intensity	Low Intensity	1153 (38.74)	5094 (46.63)	<0.001
	High Intensity	1823 (61.26)	5830 (53.37)	
Number of Attended Sessions	Low Intensity	2.36 (2.46)	2.75 (2.63)	<0.001
	High Intensity	4.64 (5.30)	4.65 (5.40)	
*End of treatment Factors*				
PHQ-9 Score		11.58 (7.06)	9.26 (6.21)	<0.001
GAD-7 Score		10.32 (6.07)	8.41 (5.48)	<0.001
Reliable Recovery	No	3142 (68.3)	8646 (55.2)	<0.001
	Yes	1459 (31.7)	7026 (44.8)	
Reliable Improvement	No	1893 (41.1)	4799 (30.6)	<0.001
	Yes	2715 (58.9)	10886 (69.4)	
Reliable Deterioration	No	4151 (90.1)	14581 (93)	<0.001
	Yes	457 (9.9)	1104 (7)	
Attrition	No	2177 (53.8)	9311 (65.9)	<0.001
	Yes	1867 (46.2)	4817 (34.1)	
Proportion of offer sessions attended		0.75 (0.19)	0.80(0.17)	<0.001
Employment Status	Employed, Vocational Training or Student	1705(37.5)	14391 (92.5)	<0.001
	Unemployed	2847 (62.5)	1170 (7.5)	

### The association between NEET status and treatment outcomes

In unadjusted models, there was evidence that both reliable recovery and reliable improvement in symptoms were less likely among those who were NEET relative to those who were not NEET (Table 3). The gap in proportions of those who were and were not NEET that reliably recovered at the end of treatment grew in the months of the COVID-19 pandemic in 2020. The difference between the groups was approximately 9–10% in 2018 and 2019 but was approximately 18% in 2020. The magnitude of the effects was reduced when adjusting for baseline clinical factors, but in the fully adjusted models those who were NEET appeared to have approximately two-thirds the odds of reliable recovery and reliable improvement relative to their not NEET peers (Table 3). Those who were NEET were also more likely to experience a reliable deterioration (worsening) of symptoms pre-post treatment. In the fully adjusted models, those who were NEET had approximately 1.3 times the odds of attrition. In line with this, in the fully adjusted models, on average those who were NEET attended between three and four per cent fewer sessions of those that were booked with their therapist, compared to those that were not NEET (Table 3).

**Table 3.** Associations between each outcome and NEET status, crude and adjusted for increasing numbers of potential confounding factors

Outcome Variable	Model					
	Crude Effects	Adjusted for Clinical Factors^a^	Additionally Adjusted for socio-demographics^b^	Additionally adjusted for treatment factors^c^	Additionally adjusted for cohort factors^d^	Additionally adjusted for service factors^e^
	OR (95% CI)					
Reliable Recovery	0.57 (0.53–0.61)	0.66 (0.62–0.71)	0.66 (0.61–0.71)	0.69 (0.64–0.74)	0.70 (0.65–0.75)	0.67 (0.63–0.73)
Reliable Improvement	0.63 (0.59–0.68)	0.65 (0.60–0.69)	0.64 (0.61–0.71)	0.67 (0.63–0.73)	0.69 (0.64–0.74)	0.68 (0.63–0.73)
Reliable Deterioration	1.45 (1.30–1.63)	1.48 (1.31–1.66)	1.49 (1.32–1.68)	1.44 (1.27–1.63)	1.39 (1.23–1.58)	1.41 (1.25–1.60)
Attrition	1.67 (1.56–1.79)	1.49 (1.39–1.60)	1.47 (1.36–1.58)	1.37 (1.25–1.49)	1.36 (1.25–1.49)	1.31 (1.19–1.43)
	Beta (95% CI), *p*-value					
Engagement	−0.05 (−0.06–−0.04), *p* < 0.001	−0.04 (−0.05–−0.04), *p* < 0.001	−0.04 (−0.05– −0.04), *p* < 0.001	−0.03 (−0.04– −0.03), *p* < 0.001	−0.03 (−0.04–−0.03), *p* < 0.001	−0.03 (−0.04–−0.03), *p* < 0.001

a Adjusted for pre-treatment PHQ-9 scores, GAD-7 scores, W&SAS items 2-5 scores, IAPT phobias scale item scores, psychotropic medication, and diagnosis.

b Additionally adjusted for gender, age, ethnicity, IMD decile, and long-term conditions.

c Additionally adjusted for the number of LI sessions, the number of HI sessions, days between referral and assessment, days between assessment and starting treatment.

d Additionally adjusted for year and month of first appointment.

e Additionally adjusted for service data came from.

### Moderators of treatment outcomes

There was no evidence of moderation of outcomes by gender (e.g. for reliable recovery *p* = 0.800), but there was by ethnicity such that those that were NEET who identified as being of an ethnic minority group were more likely to reliably recover (*p* = 0.028), more likely to reliably improve (*p* = 0.007), and attended a higher proportion of booked appointments (*p* < 0.001) (Table 4). In addition, relative to those in the most deprived areas by indices of multiple deprivations, those in the least deprived one-third of areas in this dataset, who were NEET, were more likely to report reliable recovery (*p* = 0.012), reliable improvement (*p* = 0.006), and attended a higher proportion of the booked sessions (*p* = 0.026). There was no evidence of moderation by reporting or not reporting a comorbid long-term physical health condition (e.g. for reliable recovery *p* = 0.658). There was also no evidence of moderation by main intensity (LI or HI) of treatment (e.g. for reliable recovery *p* = 0.314). There was little evidence of moderation by the four factors identified by Clark et al. (2018) investigated here (Table 5), for example with the primary outcome: number of sessions (*p* = 0.332), number of missed appointments (*p* = 0.154), missing diagnosis (*p* = 0.415), and having a first treatment appointment within 6 weeks (*p* = 0.216). However, in the stratified analyses for every additional attended session on average, those who were NEET were more likely to reliably recover [odds ratio (OR) [95% confidence interval (CI)] = 1.10 (1.08–1.12)], reliably improve, less likely to deteriorate [OR (95% CI) = 0.94 (0.91–0.98)], and attrition was less likely, see Table 5. To further demonstrate the effect, for every three additional sessions the odds of reliable recovery for those who were NEET were considerably greater [OR (95% CI) = 1.31 (1.24–1.38)] such that attending at least nine sessions was associated with more than double the odds of reliable recovery [OR (95% CI) = 2.33(1.93–2.82)], and two-thirds the odds of deterioration [OR (95% CI) = 0.66 (0.48–0.92)], relative to attending fewer than nine sessions. Those who were NEET and missed more appointments were less likely to reliably recover OR (95% CI) = 0.95 (0.92–0.99). Those who were NEET and had a missing diagnosis code (or ‘problem descriptor’) were no more or less likely to report any of the clinical outcomes. However, attrition appeared to be less likely among those who were NEET and had a missing diagnosis code compared to those with a recorded diagnosis: OR (95% CI) = 0.68 (0.50–0.92), and those with a missing diagnostic code attended a higher proportion of booked appointments. Those who were NEET that had the first appointment within 21 days had greater odds of reliable recovery: OR (95% CI) = 1.27 (1.03–1.57).

**Table 4.** Associations between each outcome and NEET status moderated by baseline characteristic, in fully adjusted models^a^

Interaction	Outcome [OR (95%CI) unless otherwise stated]				
	Reliable recovery	Reliable improvement	Reliable deterioration	Attrition	Engagement (Beta (95%CI), *p*-value)
NEET and Female Gender	0.96 (0.82–1.13)	0.98 (0.84–1.14)	0.9 (0.70–1.17)	0.89 (0.74–1.07)	0.00 (−0.02 to 0.01), *p* = 0.475
NEET and Black or minority ethnicity	1.18 (1.02–1.37)	1.22 (1.06–1.41)	0.82 (0.65–1.04)	0.91 (0.77–1.09)	0.00 (−0.02 to 0.01), *p* < 0.001
NEET and IMD Tertile 2	0.96 (0.80–1.14)	0.93 (0.78–1.10)	1.24 (0.94–1.65)	0.99 (0.80–1.21)	0.00 (−0.02 to 0.01), *p* = 0.618
NEET and IMD Tertile 3	1.26 (1.05–1.51)	1.28 (1.07–1.53)	1.06 (0.79–1.43)	0.94 (0.76–1.15)	1.02 (1.00 to 1.03), *p* = 0.012
NEET and LTC Yes	1.05 (0.85–1.29)	1.12 (0.92–1.37)	0.81 (0.58–1.13)	0.81 (0.63–1.02)	0.00 (−0.02 to 0.01), *p* = 0.022
NEET and LTC Missing	1.14 (0.94–1.38)	1.05 (0.87–1.27)	1.16 (0.85–1.59)	0.92 (0.74–1.15)	0.00 (−0.01 to 0.02), *p* = 0.839
NEET and High Intensity Treatment	0.92 (0.77–1.09)	0.88 (0.73–1.05)	1.19 (0.90–1.58)	0.99 (0.82–1.20)	0.00 (−0.01 to 0.02), *p* = 0.529

a All models adjusted for PHQ-9 scores, GAD-7 scores, W&SAS items 2-5 scores, IAPT phobias scale item scores, psychotropic medication, diagnosis, gender, age, ethnicity, IMD decile, long-term conditions, number of LI sessions, number of HI sessions, days between referral and assessment, days between assessment and starting treatment, year and month of the first appointment, and service data came from. Items from this list were excluded if the same as or highly collinear with the moderating variable (e.g. gender, ethnicity, IMD Decile, and long-term conditions).

**Table 5.** Associations between each outcome with each potential moderator in a stratified analysis of those who were NEET only

Moderator investigated in stratified analysis	Outcome [OR (95%CI) unless otherwise stated]^a^				
	Reliable recovery	Reliable improvement	Reliable deterioration	Attrition	Engagement (Beta (95% CI), *p* value)
Number of Missed Appointments	0.95 (0.92–0.99)	0.98 (0.95–1.16)	1.08 (1.02–1.14)	1.13 (1.09–1.17)	−0.06 (−0.06 to −0.06), *p* < 0.001
Number of therapy sessions attended (per one session)	1.09 (1.07–1.11)	1.11 (1.09–1.13)	0.95 (0.92–0.98)	0.71 (0.69–0.73)	0.01 (0.01–0.01), *p* < 0.001
No recorded Diagnosis	1.10 (0.84–1.45)	1.01 (0.79–1.29)	0.86 (0.57–1.28)	0.68 (0.50–0.92)	0.04 (0.02–0.06), *p* < 0.001
Number of weeks between referral and starting treatment	0.99 (0.97–1.01)	0.99 (0.97–1.00)	0.98 (0.95–1.01)	1.00 (0.98–1.02)	0 (0–0), *p* = 0.013
Started treatment within 21 days of assessment	1.27 (1.03–1.57)	1.25 (1.02–1.53)	0.58 (0.41–0.83)	0.76 (0.60–0.96)	0.04 (0.03–0.06), *p* < 0.001

a All models adjusted for PHQ-9 scores, GAD-7 scores, W&SAS items 2-5 scores, IAPT phobias scale item scores, psychotropic medication, diagnosis, gender, age, ethnicity, IMD decile, long-term conditions, number of LI sessions, number of HI sessions, days between referral and assessment, days between assessment and starting treatment, year and month of the first appointment, and service data came from. Items from this list were excluded if the same as or highly collinear with the moderating variable (e.g. number of attended appointments, number of HI sessions, number of LI sessions, diagnosis, and days between referral and starting treatment).

### Sensitivity analyses

There were very few substantive differences when analyses were conducted using mixed-effects models for service level clustering (online Supplementary Table S2) and when conducted on observed data compared to the primary analyses with imputed data (online Supplementary Tables S3–S6). The only differences of note were that in the observed data, there appeared to be some evidence of an interaction between treatment intensity and NEET status such that those who were NEET and predominantly had HI treatment were less likely to reliably improve pre-post-treatment OR (95% CI) = 0.70 (0.56–0.87), and more likely to reliably deteriorate (1.49 (1.02–2.17)). There was also less strong evidence of an interaction between ethnicity and treatment outcomes in the observed data: OR (95% CI) for reliable recovery among BAME participants who were NEET = 1.16 (0.97–1.38).

## Discussion

Young adults who were NEET seeking psychological treatment for common mental disorders in primary care had worse treatment outcomes than young adults who were not NEET. Specifically, those who were NEET had approximately two-thirds the odds of reliable recovery, about 1.4 times the odds of reliable deterioration and about a third higher odds of attrition even after adjustment for key clinical and demographic variables. Those who were NEET and of an ethnic minority had better clinical outcomes than White young adults who were NEET, and they attended a higher proportion of sessions. Those who were NEET and lived in the least deprived areas had similar outcomes to those who were not NEET. These outcomes were considerably better than those experienced by participants that were NEET and lived in the most deprived or moderately deprived areas. There was a lack of evidence that the type of treatment (HI or LI psychological therapy) moderated outcomes for those who were NEET. Importantly, the more sessions those who were NEET had, the better their chance of good treatment outcomes became. Those who were NEET had considerably greater odds of reliable recovery and reliable improvement, and lesser odds of deterioration and attrition if they attended more sessions. For example, the odds of reliable recovery were about 1.3 times higher for each additional three sessions attended, such that attending nine or more sessions was associated with more than double the odds of reliable recovery relative to attending fewer sessions. Those who were NEET and missed (cancelled or did not attend) more appointments had worse treatment outcomes, higher odds of attrition and worse engagement than those who missed fewer sessions. Waiting fewer days between referral (or registering with the services) and having a first appointment was associated with considerably better outcomes. Although, contrary to expectations, those who were NEET and had no recorded diagnosis did not appear to have worse outcomes than those with a diagnosis, and there was some evidence that they were likely to attend more sessions, and that attrition was less likely. The reasons for these effects could not be determined with the available data.

### Limitations

In this clinical cohort study, there were very high rates of data completion both at baseline and post-treatment, reducing some sources of bias. By drawing on routinely collected clinical data in a group of high-volume services a large cohort of young adults was studied, providing more accurate estimates of effects than has been possible with many (smaller) studies of those that are NEET to date. However, there were a number of limitations. The cohort here had at least two treatment sessions which might have introduced selection biases as those who are NEET with better prognoses might have been more likely to attend the services than those with poorer prognoses. It was beyond the scope of the present study to investigate the reasons for not attending the services among young adults, but future research into this topic could be particularly valuable. In addition, studies might investigate any differential outcomes between those who are NEET and have or have not had prior mental health care, for example in child and adolescent mental health services, and how such care may have impacted expectations of care in adult services.

Although adjustments were made for a number of confounding factors, including those found to be associated with outcomes in similar cohorts in the past (Delgadillo, Moreea, & Lutz, 2016; Finegan, Firth, & Delgadillo, 2020; Firth, Delgadillo, Kellett, & Lucock, 2020; Saunders et al., 2019; Saunders, Buckman, & Pilling, 2020; Saunders, Cape, et al., 2020), we cannot rule out residual confounding, or confounding by variables not available here, such as information on personality difficulties or treatment expectancy (Delgadillo et al., 2016; Goddard, Wingrove, & Moran, 2015; Mars et al., 2021). In addition, it has been argued that those who are NEET are less likely to live in stable housing than their not NEET peers (Robert et al., 2019), this may have contributed to their ability to attend and engage with services, data were not available on the length of housing occupancy here. Further, there might be a degree of reverse causality for example the ‘Healthy Worker Effect’ (Li & Sung, 1999) which could explain some of the disparity in clinical outcomes between those who were and were not NEET. Adjustments for long-term health conditions had minimal impact on the findings here, however it was not possible to address this fully with the data available in this study.

Eight IAPT services in the greater London area provided data as part of the NCEL network, however, the generalisability of the findings both to those outside of London and those in other clinical settings may be questioned, particularly for those findings related to social deprivation at the area-level. That notwithstanding, the disparities in deprivation across and within the areas covered by the eight services are large, with greater variability in these factors than might be found in services operating outside of London. It is also noteworthy that participants in this study most often attended fewer treatment sessions than are recommended in clinical guidelines, although this is a common phenomenon in routine clinical practice (e.g. Community and Mental Health team, 2019), it might have affected the generalisability of findings here. In addition, one of the outcomes studied here was the proportion of booked appointments attended, and we have taken this as a proxy for engagement in treatment. However, the accuracy of this as a proxy for engagement is questionable, with no information about the degree of learning occurring within the treatment sessions or of the amount of between-session work (‘homework’) conducted by patients outside of the therapy sessions. These factors are thought to be central to the outcomes achieved in many psychological therapies, particularly those that are based on cognitive behaviour therapy (Cuijpers, Karyotaki, Reijnders, & Huibers, 2018; Ewbank et al., 2020; Karyotaki et al., 2018, 2017; Mohr et al., 2012), which is the predominant modality used in IAPT services. Another outcome addressed was attrition, it might be considered circular to investigate the association between the number of attended sessions and attrition, however, given the nature of treatment in IAPT services, including variable treatment lengths and stepping up and down between high and low intensities, this was not the case here. Indeed, the maximum number of attended sessions for any participant prior to attrition was 26, and the minimum for any participant that completed therapy was two.

### Implications

Young adults who are not in employment, education or training (NEET) are known to have poorer mental health than peers in employment, education or training, and to be at greater risk of social health problems (Bäckman & Nilsson, 2016; Gutiérrez-García et al., 2017; McDaid et al., 2019; O'Dea et al., 2016). There has recently been great concern that those who were NEET prior to the COVID-19 pandemic or those who are now NEET as a result of the pandemic are at risk of poor mental health outcomes (Fancourt, Steptoe, & Bu, 2020; Holmes et al., 2020; Power et al., 2020). This has led to suggestions of increasing access to psychological therapies specifically for young adults affected by the pandemic (Gunnell et al., 2020; Kola, 2020; Liu, Stevens, Conrad, & Hahm, 2020; Zhou, Liu, Xue, Yang, & Tang, 2020). The findings of the present study support such assertions of those who are NEET (approximately 59% of those who were NEET experienced a reliable improvement in symptoms in this study). However, the clinical outcomes they achieved appear to be worse than those of young adults who were employed or in education. This effect was more extreme in the months of the COVID-19 pandemic in 2020 with the difference in the rate of those who were and those who were not NEET reliably recovering growing from approximately 9–10% in 2018 and 2019, to 18% in 2020, suggesting additional adaptations may be required to optimise treatment outcomes for this population. Programs that seek to support young adults to stay in education, training, or employment, or those aimed at helping those who are NEET back into such settings, may be particularly important (Mawn et al., 2017; Moore et al., 2017; Richter & Hoffmann, 2019). Whether such programs are effective at improving the engagement and clinical outcomes of those who are NEET in primary care mental health services is a question for future studies. In addition, evaluations of programs to address digital inequalities affecting access to care for those who are NEET during the pandemic and beyond may also be informative, particularly if therapy delivered remotely is still necessary or a preferred option for some patients (Buckman, Saunders, Leibowitz, & Minton, 2021; Cromarty, Gallagher, & Watson, 2020). Those who were NEET and lived in more socially deprived areas and those from White ethnic backgrounds appeared to be most at risk of poor outcomes in this study. With the data available in this study we were not able to determine why this was the case. It might therefore be helpful to consider additional research including studies focussed on intersectionality to understand the nature of these disparities and what additional support might be offered to improve engagements and outcomes for those who are NEET.

On the basis of the stratified analyses here, it would appear that starting treatment sooner, and supporting those who are NEET to attend more sessions, in particular, might be effective ways of improving their clinical outcomes. Interventions to reduce missed appointments by making changes to the organisational systems for booking appointments and sending patients reminders of their appointments appear to have been beneficial elsewhere (Aggarwal, Davies, & Sullivan, 2016; Behavioural Insights Team, 2010; Margham, Williams, Steadman, & Hull, 2021). Investigating ways to apply such learning to best meet the needs of those who are NEET could be informative. This might include qualitative interviews, outreach work and co-creation of programs with those who are NEET, thereby ensuring buy-in from those that are underserved by mental health services, in particular those in more socially deprived areas. It was notable that fewer young adults that were NEET started treatment, and fewer had predominantly LI treatments. It is often the case in primary care mental health services that LI treatments have a shorter waiting list than HI ones, and as such it would also be informative to test the effect of providing LI treatment, initially or wholly, to those who are NEET with the aim of them starting treatment sooner, and stepping up to HI treatment once available, if appropriate.

## Supplementary material

For supplementary material accompanying this paper visit https://doi.org/10.1017/S0033291721004773. click here to view supplementary material
