# Supplementary material for: Understanding the psychological therapy treatment outcomes for young adults who are not in education, employment, or training (NEET), moderators of outcomes, and what might be done to improve them
Source: Psychol Med. 2021 Nov 25;53(7):2808–19. doi: 10.1017/S0033291721004773 (PMC10235648; doi:10.1017/S0033291721004773)
Supplement: Supplementary file 1 [file S0033291721004773sup.zip › S0033291721004773sup001.docx]

**Supplementary Figure 1.** Participant Flow with Reasons for Exclusions

**Included in Analyses**

N=20,293

**Entering Treatment**

n=301,933

**Excluded**

Under 18 years old (n=3,879)

25 years old or over (n=253,512)

In voluntary employment only (n=28)

Only pre-treatment data (n=2,425)

Not at caseness pre-treatment (n=3,984)

Non-IAPT Diagnosis (n=707)

Only one treatment session (n=15,700)

Still in treatment (n=1405)
