# Supplementary material for: Understanding the psychological therapy treatment outcomes for young adults who are not in education, employment, or training (NEET), moderators of outcomes, and what might be done to improve them
Source: Psychol Med. 2021 Nov 25;53(7):2808–19. doi: 10.1017/S0033291721004773 (PMC10235648; doi:10.1017/S0033291721004773)
Supplement: Supplementary file 1 [file S0033291721004773sup.zip › S0033291721004773sup002.docx]

# Supplementary Materials to Understanding the Psychological Therapy Treatment Outcomes for Young Adults who are Not in Education, Employment, or Training (NEET), moderators of outcomes, and what might be done to improve them

## Content

1. Information on the Anxiety Disorder Specific Measures used by IAPT services.
2. Results of sensitivity analyses: i) using multi-level models with random effects for service level clustering; ii) on observed data only with cases dropped by list-wise deletion due to missing data.

### Supplementary Methods

**Anxiety Disorder Specific Measures used by IAPT services.**

**Supplementary Table 1. Recommended ADSMs (adapted from NHS Digital** (NHS Digital, 2016)**).**

| **Problem descriptor** | **Recommended ADSM** | **Threshold for caseness** | **Threshold for reliable change** |
| --- | --- | --- | --- |
| Agoraphobia | Mobility Inventory(Chambless, Caputo, Jasin, Gracely, & Williams, 1985) | 2.3 (for version 1.5) | 0.73 (for version 1.5) |
| Health anxiety | Health Anxiety Inventory (Salkovskis, Rimes, Warwick, & Clark, 2002) | 18 | 4 |
| Obsessive compulsive disorder (OCD) | Obsessive Compulsive Inventory (Foa, Kozak, Salkovskis, Coles, & Amir, 1998) | 40 | 32 |
| Panic disorder | Panic Disorder Severity Scale (Shear et al., 2001) | - | - |
| Post-traumatic stress disorder (PTSD) | Impact of Events Scale (IES-R) (Creamer, Bell, & Failla, 2003) | 33 | 9 |
| Social anxiety disorder | Social Phobia Inventory(Connor et al., 2000) | 19 | 10 |

Supplementary Table 1 presents the recommended Anxiety Disorder Specific Measures (ADSMs) used in IAPT services for each anxiety disorder problem descriptor. The table also includes the threshold which indicates clinical caseness on each measure as well as the number of points of change on the measure which are used to indicate reliable change (improvement/deterioration). Note that the PDSS does not have a threshold for caseness or reliable change and instead the GAD-7 is used in the calculation of key IAPT outcomes for these individuals.

### Sensitivity Analyses

**Supplementary Table 2.** Associations between each outcome and NEET status, with and without mixed effects modelling to account for potential service level clustering^¶^, using observed data only.

|  | **Model** | |
| --- | --- | --- |
| **Outcome Variable** | **Original Analysis** | **Mixed Effects Model Analysis** |
|  | **OR(95%CI)** | |
| Reliable Recovery | 0.68(0.62 to 0.74) | 0.69(0.63 to 0.75) |
| Reliable Improvement | 0.68(0.62 to 0.74) | 0.68(0.63 to 0.74) |
| Reliable Deterioration | 1.40(1.20 to 1.60) | 1.40(1.22 to 1.61) |
| Attrition | 1.30(1.16 to 1.43) | 1.28(1.15 to 1.42) |
|  | **Beta(95%CI), p-value** | |
| Engagement | -0.03(-0.03 to -0.02), p<.001 | -0.03(-0.04 to -0.02), p<.001 |

^¶^All models adjusted for PHQ-9 scores, GAD-7 scores, W&SAS items 2-5 scores, IAPT phobias scale item scores, psychotropic medication, diagnosis, gender, age, ethnicity, IMD decile, long-term conditions, number of LI sessions, number of HI sessions, days between referral and assessment, days between assessment and starting treatment, year and month of first appointment, and service data came from.

**Supplementary Table 3.** Associations between each outcome and NEET status, crude and adjusted for increasing numbers of potential confounding factors, using observed data only.

|  | **Model** | | | | | |
| --- | --- | --- | --- | --- | --- | --- |
| **Outcome Variable** | **Crude Effects** | **Adjusted for Clinical Factors**^†^ | **Additionally Adjusted for socio-demographics**^‡^ | **Additionally adjusted for treatment factors**^§^ | **Additionally adjusted for cohort factors^⸹^** | **Additionally adjusted for service factors**^¶^ |
|  | **OR(95%CI)** | | | | | |
| Reliable Recovery | 0.57(0.53 to 0.61) | 0.68(0.62 to 0.73) | 0.67(0.62 to 0.73) | 0.7(0.64 to 0.76) | 0.71(0.65 to 0.77) | 0.68(0.62 to 0.74) |
| Reliable Improvement | 0.63(0.59 to 0.68) | 0.64(0.59 to 0.7) | 0.64(0.62 to 0.73) | 0.67(0.62 to 0.73) | 0.69(0.63 to 0.75) | 0.68(0.62 to 0.74) |
| Reliable Deterioration | 1.45(1.29 to 1.62) | 1.47(1.27 to 1.68) | 1.48(1.27 to 1.69) | 1.42(1.22 to 1.62) | 1.38(1.18 to 1.58) | 1.4(1.2 to 1.6) |
| Attrition | 1.66(1.54 to 1.78) | 1.5(1.37 to 1.62) | 1.47(1.34 to 1.59) | 1.39(1.24 to 1.53) | 1.34(1.2 to 1.48) | 1.3(1.16 to 1.43) |
|  | **Beta(95%CI), p-value** | | | | | |
| Engagement | -0.05(-0.06 to -0.04), p<.001 | -0.04(-0.05 to -0.03), p<.001 | -0.04(-0.05 to -0.03), p<.001 | -0.03(-0.04 to -0.02), p<.001 | -0.03(-0.03 to -0.02), p<.001 | -0.03(-0.03 to -0.02), p<.001 |
| ^†^adjusted for pre-treatment PHQ-9 scores, GAD-7 scores, W&SAS items 2-5 scores, IAPT phobias scale item scores, psychotropic medication, and diagnosis. ^‡^additionally adjusted for gender, age, ethnicity, IMD decile, and long-term conditions; ^§^ additionally adjusted for number of LI sessions, number of HI sessions, days between referral and assessment, days between assessment and starting treatment; **^⸹^**additionally adjusted for year and month of first appointment; ^¶^additionally adjusted for service data came from. | | | | | | |

**Supplementary Table 4.** Associations between each outcome and NEET status moderated by baseline characteristic, in fully adjusted models^¶^, using observed data only.

|  | **Outcome (OR (95%CI) unless otherwise stated)** | | | | |
| --- | --- | --- | --- | --- | --- |
| **Interaction** | **Reliable Recovery** | **Reliable Improvement** | **Reliable Deterioration** | **Attrition** | **Engagement (Beta (95%CI) , p-value)** |
| NEET and Female Gender | 0.96(0.8 to 1.15) | 0.94(0.78 to 1.12) | 1.05(0.77 to 1.42) | 0.95(0.76 to 1.18) | -0.01(-0.02 to 0), p=.189 |
| NEET and Black or minority ethnicity | 1.16(0.97 to 1.38) | 1.19(1 to 1.42) | 0.9(0.67 to 1.2) | 0.87(0.7 to 1.07) | 0.03(0.02 to 0.04), p<.001 |
| NEET and IMD Tertile 2 | 0.95(0.78 to 1.17) | 0.95(0.78 to 1.16) | 1.09(0.78 to 1.51) | 1(0.78 to 1.28) | 0.01(-0.01 to 0.02), p=.369 |
| NEET and IMD Tertile 3 | 1.28(1.04 to 1.57) | 1.27(1.03 to 1.56) | 0.89(0.63 to 1.27) | 0.94(0.73 to 1.21) | 0.01(-0.01 to 0.02), p=.238 |
| NEET and LTC Yes | 1.09(0.86 to 1.37) | 1.28(1.02 to 1.61) | 0.79(0.54 to 1.16) | 0.81(0.61 to 1.07) | 0.01(0 to 0.03), p=.126 |
| NEET and LTC Missing | 1.1(0.88 to 1.38) | 1.06(0.85 to 1.32) | 1.13(0.78 to 1.63) | 0.83(0.63 to 1.09) | 0(-0.02 to 0.02), p=.949 |
| NEET and High Intensity Treatment | 0.83(0.68 to 1.02) | 0.7(0.56 to 0.87) | 1.49(1.02 to 2.17) | 0.99(0.77 to 1.27) | 0.01(0 to 0.02), p=.146 |

^¶^All models adjusted for PHQ-9 scores, GAD-7 scores, W&SAS items 2-5 scores, IAPT phobias scale item scores, psychotropic medication, diagnosis, gender, age, ethnicity, IMD decile, long-term conditions, number of LI sessions, number of HI sessions, days between referral and assessment, days between assessment and starting treatment, year and month of first appointment, and service data came from. Items from this list were excluded if the same as or highly collinear with the moderating variable (e.g. gender, ethnicity, IMD Decile, and long-term conditions).

**Supplementary Table 5.** Associations between each outcome with each potential moderator in stratified analysis of those who were NEET only, using observed data only.

|  | **Outcome (OR (95%CI) unless otherwise stated)** ^¶^ | | | | |
| --- | --- | --- | --- | --- | --- |
| **Moderator investigated in stratified analysis** | **Reliable Recovery** | **Reliable Improvement** | **Reliable Deterioration** | **Attrition** | **Engagement (Beta (95%CI) , p-value)** |
| Number of Missed Appointments | 0.95(0.92 to 0.99) | 0.98(0.95 to 1.16) | 1.08(1.02 to 1.14) | 1.13(1.09 to 1.17) | -0.06(-0.06 to -0.06), p<.001 |
| Number of therapy sessions attended (per one session) | 1.09(1.07 to 1.11) | 1.11(1.09 to 1.13) | 0.95(0.92 to 0.98) | 0.71(0.69 to 0.73) | 0.01(0.01 to 0.01), p<.001 |
| No recorded Diagnosis | 1.10(0.84 to 1.45) | 1.01(0.79 to 1.29) | 0.86(0.57 to 1.28) | 0.68(0.50 to 0.92) | 0.04(0.02 to 0.06), p<.001 |
| Number of weeks between referral and starting treatment | 0.99(0.96 to 1.01) | 0.98(0.96 to 1.00) | 0.98(0.95 to 1.02) | 1.00(0.97 to 1.03) | 0.00(0.00 to 0.00), p=.279 |
| Started treatment within 21 days of assessment | 1.27(1.03 to 1.57) | 1.25(1.02 to 1.53) | 0.58(0.41 to 0.83) | 0.76(0.60 to 0.96) | 0.04(0.03 to 0.06), p<.001 |

^¶^All models adjusted for PHQ-9 scores, GAD-7 scores, W&SAS items 2-5 scores, IAPT phobias scale item scores, psychotropic medication, diagnosis, gender, age, ethnicity, IMD decile, long-term conditions, number of LI sessions, number of HI sessions, days between referral and assessment, days between assessment and starting treatment, year and month of first appointment, and service data came from. Items from this list were excluded if the same as or highly collinear with the moderating variable (e.g. number of attended appointments, number of HI sessions, number of LI sessions, diagnosis, and days between referral and starting treatment).

**References.**

Chambless, D. L., Caputo, G. C., Jasin, S. E., Gracely, E. J., & Williams, C. (1985). The Mobility Inventory for Agoraphobia. *Behaviour Research and Therapy*, *23*(1), 35–44. https://doi.org/10.1016/0005-7967(85)90140-8

Connor, K. M., Davidson, J. R. T., Churchill, L. E., Sherwood, A., Weisler, R. H., & Foa, E. (2000). Psychometric properties of the Social Phobia Inventory (SPIN). *The British Journal of Psychiatry*, *176*(4), 379–386. https://doi.org/10.1192/bjp.176.4.379

Creamer, M., Bell, R., & Failla, S. (2003). Psychometric properties of the Impact of Event Scale - Revised. *Behaviour Research and Therapy*, *41*(12), 1489–1496. https://doi.org/10.1016/j.brat.2003.07.010

Foa, E. B., Kozak, M. J., Salkovskis, P. M., Coles, M. E., & Amir, N. (1998). The validation of a new obsessive-compulsive disorder scale: The obsessive-compulsive inventory. *Psychological Assessment*, *10*(3), 206–214. https://doi.org/10.1037/1040-3590.10.3.206

NHS Digital. (2016). *Psychological Therapies, Annual Report on the use of IAPT services: England 2015-16*.

Salkovskis, P. M., Rimes, K. A., Warwick, H. M. C., & Clark, D. (2002). The Health Anxiety Inventory: development and validation of scales for the measurement of health anxiety and hypochondriasis. *Psychological Medicine*, *32*(5), 843–853.

Shear, M. K., Rucci, P., Williams, J., Frank, E., Grochocinski, V., Vander Bilt, J., … Wang, T. (2001). Reliability and validity of the Panic Disorder Severity Scale: Replication and extension. *Journal of Psychiatric Research*, *35*(5), 293–296. https://doi.org/10.1016/S0022-3956(01)00028-0
